# Research and Industry for Human Health Enabled by Detergent Chemistry

**DOI:** 10.1002/cplu.202500690

**Published:** 2025-12-02

**Authors:** Leonhard H. Urner

**Affiliations:** ^1^ Department of Chemistry and Chemical Biology TU Dortmund University Dortmund Germany

**Keywords:** chemistry, detergent, drug discovery, health, hygiene

## Abstract

Research and applications relevant to human health are enabled by detergent chemistry. A multifaceted overview of this field is yet missing. To close this gap, this topical collection provides an overview of recent advances in detergent chemistry covering progress in synthesis, supramolecular characterization, and application. Our collection shows that detergent chemists operate usually interdisciplinary. Connecting molecular structures of detergents with properties relevant to applications is at the center of scientific exploitation. Detergent chemists deliver solutions to research and industry that aim at securing well‐being, hygiene, and new pharmaceuticals.

## Graphical abstract

Detergent chemistry addresses urgent questions in chemical science and is pivotal to progress for human health. Guest Editor Dr. Leonhard H. Urner introduces the ChemPlusChem Special Collection “Detergent Chemistry” and provides an overview about its contents.

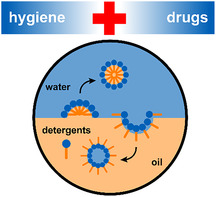

1

Detergent chemistry is making significant and widespread contributions to human health. Be it for cleaning [1], disinfection [2], or drug discovery [3], detergents act as truly magical chemicals. Like molecular diplomats, they make things miscible that would normally not mix, like oil and water (Figure 1). This fundamental property enables applications that contribute to human health in daily life, industry, and academia [4]. The ubiquitous use of detergents makes it a popular and challenging field. To support interdisciplinary exchange and awareness, we assembled articles that highlight recent advances in detergent chemistry from multifaceted views.

**FIGURE 1:**
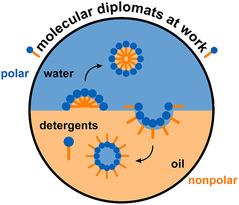
Detergents enable applications by initiating miscibility. Schematic showing detergents interacting with interfaces of immiscible liquids to initiate emulsification, i.e., a key to applications.

Detergents secure well‐being and health in domestic settings. The potential health benefits enabled by detergents for the public, as researched in academia, remain largely unexplored. The price limits detergent selection in the optimization of mass market products. The industry focuses on the interplay between detergent chemistry, application parameters, and consumer benefits. To exemplify scientific exploitation in this timely research area, Bockmühl et al*.* provide an overview about optimizing dishwashing performance with detergent chemistry and dishwasher program structures (Figure 2).

**FIGURE 2:**
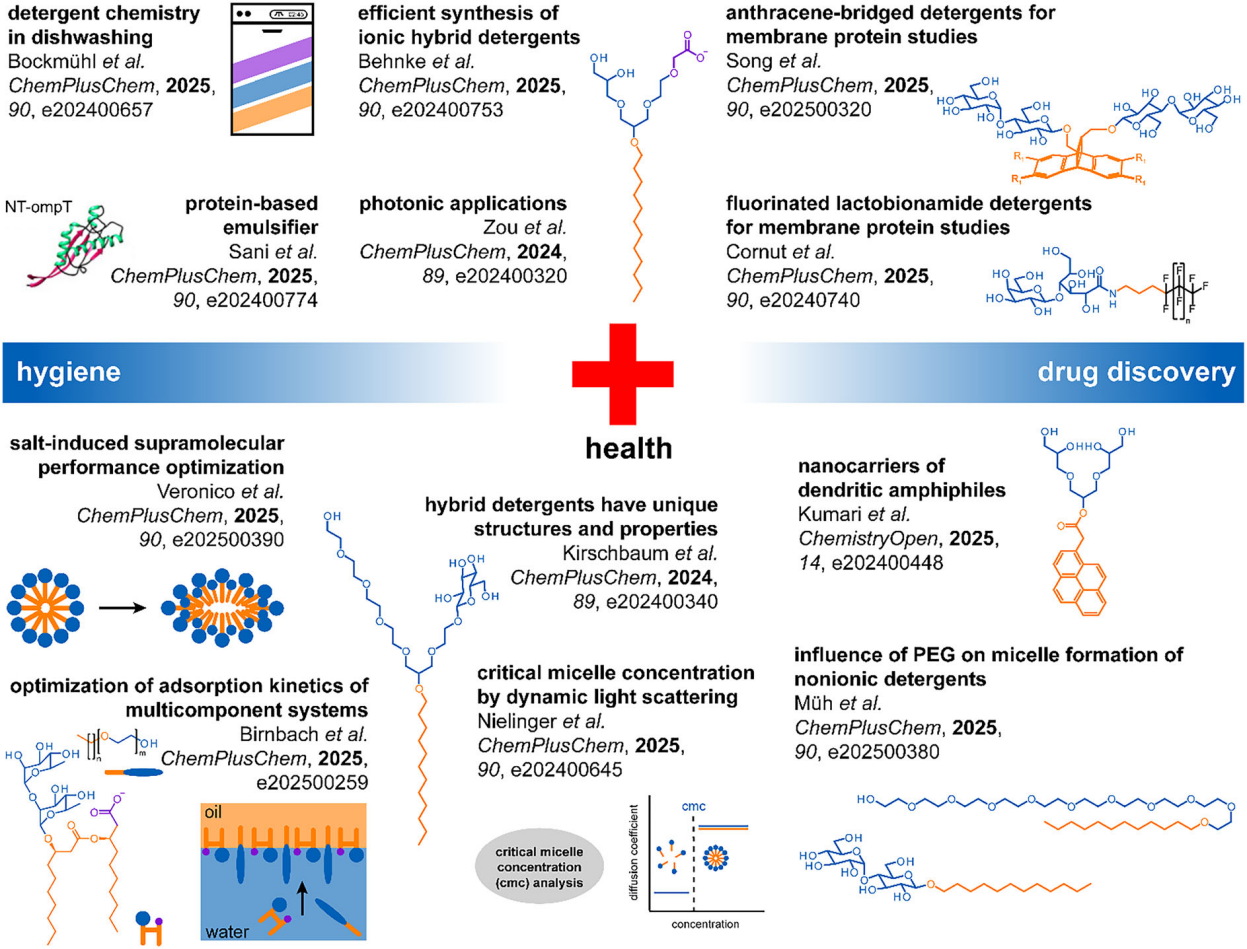
Solutions delivered to research and industry by detergent chemistry with relevance to human health. Overview of topics and articles published in the Topical Collection “Detergent Chemistry” in ChemPlusChem that are important to hygiene and drug discovery.

Detergents enable many applications through a process called emulsification. Key to this is the adsorption of detergents to interfaces between immiscible liquids (Figure 1). Adsorption kinetics of multicomponent systems are complex, which can hamper rational optimizations. To delineate the role of detergent structure from coingredients, Birnbach et al*.* showed an experimental framework to tailor the adsorption kinetics of technical ethoxylates with rhamnolipids (Figure 2). Bottom‐up approaches like this will continue in facilitating a rational optimization of complex detergent formulations.

In addition to hygiene, detergents enable drug discovery (Figure 2) [3]. The ability of detergents to form micelles in water is pivotal to experimental success. Micelles serve as nanometer‐sized carriers that can solubilize nonpolar substances in water, like drugs and proteins. To do so, detergents are handled above their minimal concentration required to form micelles (cmc). The cmc depends on the structure of detergents and solvents. Cmc values are often determined in water and correlated with experimental observations in more complex buffers. To support structure–property studies under realistic conditions, Nielinger et al*.* established a dynamic light scattering method to determine cmc values in water and buffer (Figure 2).

Veronico et al*.* demonstrate the general relevance of such approaches (Figure 2). The authors reported that the addition of salt to mixed micellar systems can change aggregate morphologies from spherical to bicelle‐like. This change contributed to the formation of transient supramolecular particle networks with increased colloidal stability, viscoelasticity, and oil‐uptake efficiency. Müh et al*.* provided fundamental insights into the roles of polyethylene glycols in the cmc of mixed micellar systems. For example, polyethylene glycols stabilize monomers of n‐dodecyl‐β‐D‐maltoside and octaethyleneglycol dodecyl ether to the same extent. This finding may support a better understanding of the transformation from type II to type I crystals in membrane protein crystallography. Understanding the correlation between structure, property, and product performance will simplify the optimization of detergent formulations in applications.

The art of designing and synthesizing detergents in academia delivers starting points for progress in drug discovery. Behnke et al*.* reported synthetic approaches to fuse ionic and nonionic detergent headgroups into hybrid detergents (Figure 2). These findings deliver new directions in the search for tools for structure‐based drug discovery and antibacterials. A key question left open about hybrid detergents was whether their chemical properties are unique or just average to their parent detergents. To close this gap, Kirschbaum et al*.* employed gas‐phase infrared spectroscopy (Figure 2). Hybrid detergents turned out to be unique molecular structures with unique properties, which implies significant innovation potential.

Approximately 60% of current drugs bind to membrane proteins [5]. Tools for drug optimization with membrane proteins are highly desired. To enable the analysis of membrane protein drug targets, new detergent architectures were established by Song et al*.* (Figure 2). The authors introduced nonionic anthracene‐bridged detergents for the solubilization and electron microscopy of G protein‐coupled receptors. Complementary, fluorinated detergents hold great potential for stabilizing intact membrane proteins. Cornut et al*.* demonstrated fluorinated lactobionamide detergents that were either effective in protein solubilization or stabilization, emphasizing the importance of detergent screening in membrane protein studies (Figure 2).

Biobased detergents hold great promise for the development of environmentally friendly methods and products [4]. Sani et al*.* demonstrated that new detergents can also be designed from proteins (Figure 2). The potential of N‐terminally truncated outer membrane protein A as a robust emulsifying agent is reported. In addition to supporting drug discovery by enabling membrane protein studies, detergents can improve drug solubility. Low drug solubility is a key hurdle in drug discovery as it causes low bioavailability [6]. Kumari et al*.* delivered new starting points in creating nanocarriers for drug delivery based on dendritic oligoglycerol amphiphiles (Figure 2). Finally, Zou et al*.* introduced multifluorinated dendrimers along with a molecular design for favorable hydrogen bond interactions with relevance for photonic applications (Figure 2).

In summary, the Topical Collection “Detergent Chemistry” clarifies a profound understanding of detergent chemistry delivers starting points for research and applications with relevance to hygiene and drug discovery (Figure 2). Advances in detergent chemistry will be pivotal in securing human health in the future.

## Conflicts of Interest

The author declares no conflicts of interest.
